# Gestational hypercalcemia: a narrative review of literature

**DOI:** 10.3389/fmed.2026.1909975

**Published:** 2026-08-19

**Authors:** Miriam Martin, Corin Heim, Martin Mueller, Markus Mohaupt

**Affiliations:** 1 Department of Medicine, Lindenhofgruppe, Bern, Switzerland; 2 Faculty of Medicine, University of Bern, Bern, Switzerland; 3 Department of Gynecology and Obstetrics, Lindenhofgruppe, Bern, Switzerland; 4 Institute of Biochemistry and Molecular Medicine, Faculty of Medicine, University of Bern, Bern, Switzerland; 5 Department of Nephrology and Hypertension, University of Bern, Bern, Switzerland

**Keywords:** calcitriol, gestational hypercalcemia, hypertension, preeclampsia, primary hyperparathyroidism

## Abstract

**Aim:**

We aim to perform a narrative literature review of gestational hypercalcemia including calcium homeostasis in pregnancy. Additionally, we demonstrate the diagnostic and therapeutic challenges and pitfalls of gestational hypercalcemia.

**Methods:**

Literature review.

**Results:**

Gestational hypercalcemia is a rare yet life-threatening condition. The presentation of gestational hypercalcemia is nonspecific and mimics common pregnancy-related symptoms contributing to missed or late diagnosis. Common morbidities include nephrolithiasis and pancreatitis in the mother and adverse obstetrical outcomes such as fetal loss, fetal growth restriction, preeclampsia and preterm delivery. Severe cases of neonatal hypocalcemia due to fetal hypoparathyroidism may lead to tetany and even death. Despite these risks, only few guidelines in the management of gestational hypercalcemia exist. Causes of gestational hypercalcemia can be divided into parathyroid hormone (PTH)-dependent versus PTH-independent disorders. PTH-dependent etiologies display increased or inappropriately high PTH levels; primary hyperparathyroidism (PHPT) and familial hypocalciuric hypercalcemia (FHH) are the main contributors. PTH-independent disorders include pseudohyperparathyroidism, malignancy, and excessive calcium intake including calcium alkali syndrome. Granulomatous diseases, cytochrome P450 family 24 subfamily A member 1 (CYP24A1) mutations, and excessive calcium intake are the main contributors. In the presence of maternal hypercalcemia, hypercalciuria is a common laboratory finding. Extended laboratory work-up is the most useful paraclinical examination in gestational hypercalcemia of unknown cause. Only FHH is usually associated with low urinary calcium excretion. Marked sarcoidosis-associated hypercalciuria in pregnancy is attributed to the increase in both renal and ectopic calcitriol production, due to pregnancy and sarcoid granulomas, respectively.

**Conclusion:**

More than 90% of cases of gestational hypercalcemia are caused by PHPT, which in turn is mostly caused (over 80%) by a single parathyroid adenoma. However, we have to consider a number of other possible etiologies. We need to consider the increased risk of maternal, neonatal, and fetal complications. The review illustrates the various clinical presentation and complex therapeutic management of gestational hypercalcemia, necessitating an interdisciplinary approach to the mother and child.

## 1 Introduction

The clinical presentation of gestational hypercalcemia is nonspecific and mimics common pregnancy-related symptoms, resulting in missed or late diagnosis. Common morbidities include nephrolithiasis and pancreatitis in the mother, and adverse obstetrical outcomes in the fetus or pregnancy, including fetal loss, fetal growth restriction (FGR), preeclampsia (PE), and preterm delivery. Severe cases of neonatal hypocalcemia due to fetal hypoparathyroidism may lead to tetany and even death. Despite these risks, only few guidelines in the management of gestational hypercalcemia exist (1, 2).

The prevalence of gestational hypercalcemia depends on the timing of diagnosis but affects ~1% of pregnancies (3–5). Most cases of gestational hypercalcemia are parathyroid hormone (PTH)-dependent, with more than 90% of those cases arising from primary hyperparathyroidism (PHPT). They are associated with increased risk of perinatal morbidity and mortality (6). Notably, symptoms such as nausea or fatigue are often misinterpreted as being related to pregnancy itself resulting in a delay of the diagnosis. In the following, we discuss the most common etiologies of gestational hypercalcemia.

## 2 Physiological changes in calcium metabolism during pregnancy

**Figure 1:**
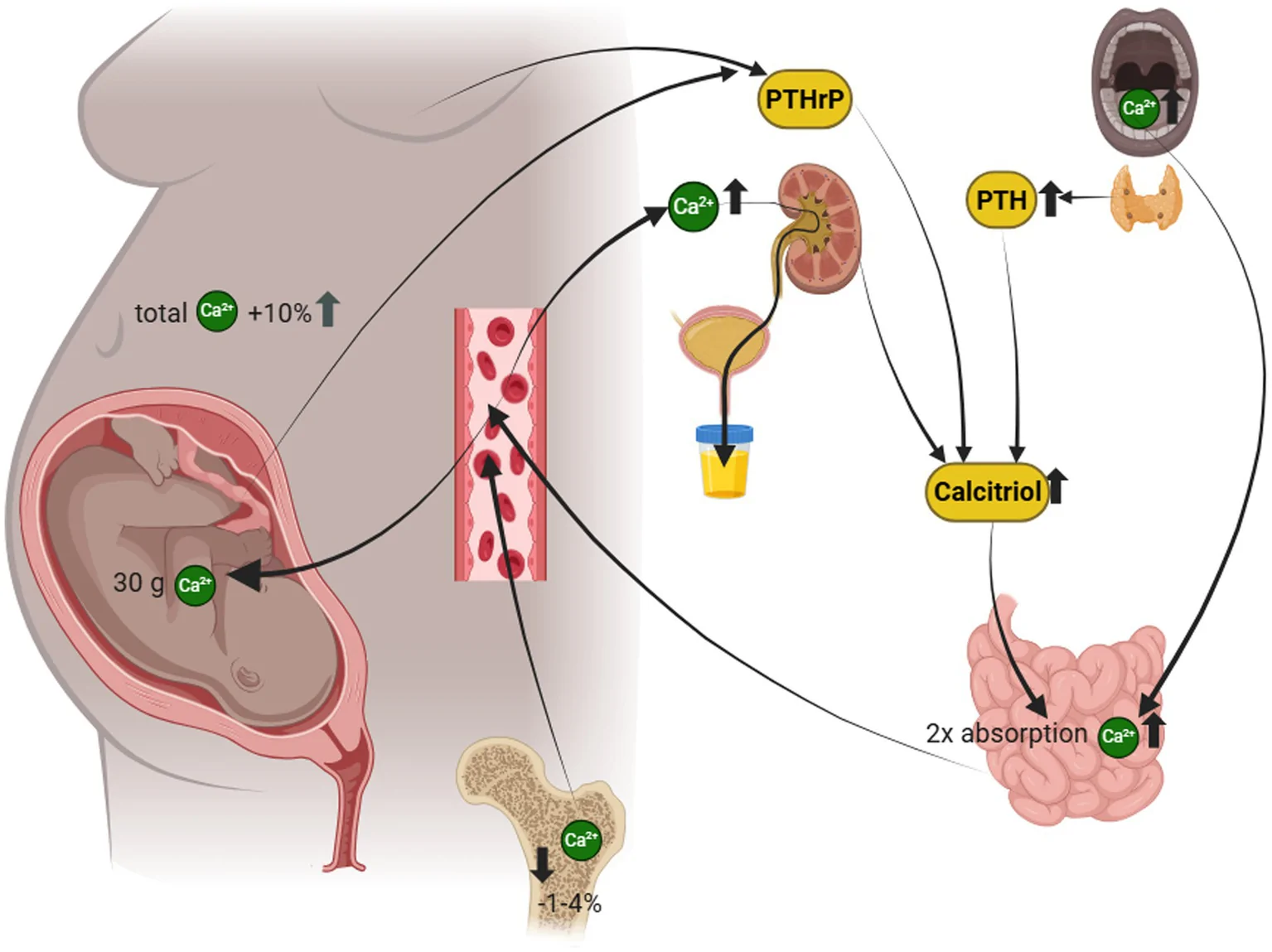
Physiological changes in calcium metabolism in pregnancy. PTHrP is primarily produced and secreted by the placenta and breasts, with its release being independent of calcium (Ca^2+^) levels. It stimulates the placental calcium transfer to the fetus and promotes osteoblast proliferation. PTHrP, PTH, and the kidneys convert the inactive 25(OH)D into its active form calcitriol increasing its levels by two to three times and resulting in a higher intestinal calcium absorption. Additionally, calcium intake in pregnancy is increased to meet the higher demands. PTHrP: parathyroid hormone related protein; PTH: parathyroid hormone.

During pregnancy, the maternal body undergoes several physiological adaptations and the growing fetus requires increasing amounts of calcium, leading to changes in calcium-phosphate metabolism. We summarized physiological changes in Figure 1. Briefly, ~99% of the human calcium is stored in the skeleton as the hydroxyapatite, a calcium phosphate salt, and in the serum ~40% of the calcium is bound to albumin and fetuin A with additional 10% complexed with anions such as citrate and bicarbonate. The remaining half of the serum calcium is metabolically available in a free ionic form (7). In pregnancy, intravascular plasma volume expansion (dilution) contributes to hypoalbuminemia resulting in a decrease of maternal total calcium levels by approximately 10% (8, 9). However, ionized (free) and albumin-adjusted calcium (= corrected calcium = measured total calcium [mmol/L] − 0,025 × albumin [g/L] + 1) remain unchanged (9). Therefore, albumin-adjusted calcium should be used for diagnostic and therapeutic decisions during pregnancy (1). The rise in intestinal calcium absorption and the glomerular hyperfiltration that occur physiologically as a result of volume expansion in pregnancy lead to an increase of up to 46% in 24-h urinary calcium excretion during the third trimester (10). The postprandial urinary calcium excretion doubles, but it remains within the normal range in the fasting state. This condition is called absorptive hypercalciuria (1) and is a risk factor for nephrolithiasis (7).

A full-term neonate contains approximately 30 grams of calcium and fetal skeletal development occurs (about 80%) within the third trimester, requiring a daily calcium transfer of 300 mg from the mother to the fetus between gestational week (GW) 35 and 40 (9). Calcium ions are actively transported from the maternal to the fetal circulation, resulting in relative fetal hypercalcemia (10). Consequently, ionized calcium levels in the fetus are 0.25–0.5 mmol/L higher than those in the mother (11). Notably, both serum phosphate and magnesium levels remain unchanged in pregnant women (9). In order to meet the fetal calcium demand, the intestinal calcium absorption in the mother doubles in pregnancy, primarily driven by the rise in 1,25-dihydroxyvitamin D (1,25(OH)_2_D), the so-called active vitamin D hormone calcitriol. In this condition, up to 25% of the ingested calcium is absorbed. In consequence, an additional maternal calcium intake of 1,200 mg daily is necessary during the last trimester (9). Calcium mobilization from the maternal skeleton as a further measure to increase the available serum calcium seems to play a subordinate role and occurs mainly in the third trimester. With an adequate calcium intake, there is only a mild and reversible reduction in maternal bone density of 1–4% during pregnancy (1).

PTHrP is produced by the placenta and mammary glands under the influence of estradiol, placental lactogen, and prolactin (10). Its release is independent of calcium levels and rarely synthetized by other tissues such as the parathyroid, myometrium, or umbilical cord (9). PTHrP consists of three regions; the N-terminal domain shows homology with PTH, accounting for its PTH-like effects. The middle part stimulates the placental calcium transfer to the fetus and the C-terminal enhances osteoblast proliferation (7). In addition, PTHrP induces the renal and placental 1α-hydroxylase (encoded by cytochrome P450 family 27 subfamily B member 1 (CYP27B1) gene) leading to a two- to threefold increase of calcitriol levels during gestation (7, 9). This elevation seems to be independent of calcium metabolism, allowing a high 1,25(OH)_2_D concentration in pregnant women (12). In summary, PTHrP induces calcitriol changes and suppresses the endogenous PTH secretion (1, 9). Neither PTH nor PTHrP crosses the placental barrier. Importantly, PTHrP is synthesized by the placenta itself and PTH by the fetal parathyroid glands after the 10th GW.

Calcitriol derives from precursors of vitamin D which are orally ingested and/or produced subcutaneously upon exposure to ultraviolet light. These prehormones are hydroxylated in the liver to 25-hydroxyvitamin D (25(OH)D), also known as calcidiol. A second hydroxylation takes place in the proximal renal tubule to form calcitriol. While maternal 1,25(OH)_2_D increases significantly, maternal 25(OH)D levels remain stable during pregnancy, readily cross the placenta and have critical role for fetal vitamin D status (9, 12). The calcidiol level in the fetus is about 20% lower than in the mother (11). Fetal kidneys initiate production of calcitriol in the third trimester, as it does not cross the placenta. The first step of 1,25(OH)_2_D degradation is encoded by cytochrome P450 family 24 subfamily A member 1 (CYP24A1) gene. Thus, plasma levels of calcitriol are primarily determined by CYP27B1 and CYP24A1 which in turn are both under the influence of calcitonin (13).

Calcitonin level normally increases in pregnant women (14). It is produced not only by the parafollicular C-cells of the thyroid gland, but also by mammary and placental tissues (9). Calcitonin does not cross the placenta (14). Among others, it decreases the osteoclastic activity and may thus play a protective role in maternal bone health during pregnancy (7, 9). During gestation, active calcium transfer to the fetus—partly mediated by PTHrP but independent of maternal PTH levels—contributes to a mild suppression of the fetal parathyroid glands (11). When placental calcium transport is suddenly interrupted at birth, a physiological decrease of neonatal serum calcium occurs reaching its nadir 6–12 h postnatally. Until full recovery of the parathyroid function 7–14 days after delivery, neonatal calcium requirements are met by calcitriol-induced intestinal calcium absorption (11).

## 3 Etiologies

From a clinical perspective, gestational hypercalcemia can be classified into two primary etiologies: PTH-dependent and PTH-independent disorders. PTH-dependent causes describe an increased PTH blood level secreted by the parathyroid glands. The main contributors are PHPT and familial hypocalciuric hypocalcemia (FHH). PTH-independent disorders usually display normal or suppressed serum PTH levels and, therefore, are independent of the parathyroid glands. PTH-independent disorders include sarcoidosis and other granulomatous diseases, drug- and nutritive-induced hypercalcemia including calcium alkali syndrome (CAS), CYP24A1 mutations and PTHrP- and malignancy-related hypercalcemia. In the following, we will discuss the different etiologies in detail.

### 3.1 PTH-dependent causes of gestational hypercalcemia

The two major contributors of the PTH-dependent disorders are primary hyperparathyroidism—PHPT and familial hypocalciuric hypercalcemia—FHH.

#### 3.1.1 Primary hyperparathyroidism (PHPT)

PHPT represents the most common cause of gestational hypercalcemia accounting for over 90% of all cases (6). As PHPT results from an overproduction of PTH from parathyroid glands—hypercalcemia is a common finding. Notably, PTH has been shown to increase serum calcium by different mechanisms. PTH augments renal tubular calcium reabsorption, stimulates skeletal calcium release, and activates 1α-hydroxylase, leading to elevated 1,25(OH)_2_D levels. Through this rise in calcitriol, PTH indirectly also increases gastrointestinal calcium reabsorption. Although PHPT represents the most common cause of hypercalcemia, PHPT incidence is significantly lower in women of childbearing age compared to those aged over 60 years (8 vs. 188/100,000) (5). Only 0.8–2.1% of cases with PHPT occur in pregnant patients (15, 16) and the prevalence of hypercalcemic PHPT in pregnancy was found to be at 0.03% (5). In a recent retrospective study including more than 1.3 million deliveries, the incidence of PHPT was 2.7/100,000 birth (17). Given this low incidence as well as the physiological decrease in total serum calcium and the suppression of PTH, the diagnosis of PHPT in pregnant women is challenging and thus likely underdiagnosed (8). Confirmation of a PHPT is achieved in the presence of an increased ionized calcium in the absence of PTH suppression (9).

PHPT in gestation usually presents as a single parathyroid adenoma (in 80–89% of cases), of which 15–16% have ectopic localizations, multiple adenomas (2 or more lesions) in less than 2%, multiglandular hyperplasia in 8–15% and parathyroid carcinoma in 1–2% (18–20). In addition to the sporadic forms, parathyroid dysplasia can occur as part of a familial syndrome, including multiple endocrine neoplasia (MEN) types 1, 2A and 4, familial isolated hyperparathyroidism, or a hyperparathyroidism-jaw tumor (HPT-JT) (18, 21, 22). These diseases are associated with PHPT due to parathyroid carcinoma or multiple adenomas. Not surprisingly, only few cases of hypercalcemia in pregnant women with MEN 1 have been reported (20, 21) alike patients with gestational hypercalcemia due to HPT-JT (22, 23). As genetic causes of PHPT are overly represented in a younger population (approximately 10% of cases (13)), they should be ruled out in gestational PHPT (8).

Perinatal morbidity of gestational PHPT has been observed in up to 67% of mothers and 80% of fetuses (24) in older publications. In a recent retrospective study, the complication rate was found to be lower with 14% in women and 45% in infants (25). The most prevalent maternal complication is nephrolithiasis occurring in up to 36% of patients, followed by pancreatitis and bone disease (both about 10%) (26, 27). Notably, osteopenia may rarely lead to fractures (19, 28). Furthermore, confusion, depression, hyperemesis, renal failure, urinary tract infection, cardiac arrythmia, and peptic ulcer, have been reported (8, 9, 29). Other rare complications include cerebral venous thrombosis (30) and hypercalcemic crisis (13). Adverse obstetric outcomes include various non-specific complications, such as fetal loss, preterm delivery, FGR, congenital anomalies, and hypertensive disorders of pregnancy (17, 31–33). If gestational PHPT remains unknown or untreated, neonatal complications, such as hypocalcemia and tetany, may occur in up to 50% of infants (11, 34, 35). Notably, hypoparathyroidism in the newborn usually resolves within 3–5 months (26) but in rare cases manifests as a permanent condition (36). The highest risk of adverse outcomes carries the hypercalcemic crisis (13). Interestingly, older and more recent studies are divergent regarding the complication rate of gestational PHPT. For instance, a retrospective patient series reported a 3.5-fold increase in the risk of fetal loss, directly related to increasing maternal serum calcium levels and most evident if hypercalcemia was greater than 2.85 mmol/L (31). This elevated PHPT-related risk of fetal loss was not confirmed in later publications (5, 37). Interestingly, mild hypercalcemia (mean ± SD 2.72 ± 0.12 mmol/L) and inclusion of asymptomatic patients with just slightly elevated serum calcium (identified through a routine screening) did not impact perinatal outcomes (5).

Up to 80% of women with gestational PHPT are asymptomatic (24) and approximatively 50% of cases are diagnosed in the postpartum period or incidentally (5, 38). Therefore, milder degrees of hypercalcemic PHPT are underdiagnosed and underrepresented in older publications, resulting in an increased rate of PHPT-associated pregnancy complications (5). Importantly, the risk of adverse perinatal outcomes is related to the degree of hypercalcemia (5, 31, 35). In contrast, as an increase of intestinal calcium absorption, hypercalciuria, and mild bone resorption is present in both gestation and PHPT, gestation itself may contribute to PHPT-induced hypercalcemia (9). This could explain why previously silent or undiagnosed PHPT may become symptomatic in pregnancy.

Finally, tertiary hyperparathyroidism or better persistent hyperparathyroidism with excessive PTH secretion and subsequent hypercalcemia may occur following correction of chronic renal failure. During renal failure, reduction of 1α-hydroxylation activity leads to hypocalcemia and chronic stimulation of the parathyroid glands (secondary hyperparathyroidism). As hyperplasia is induced and potentially autonomy of the parathyroid tissue with loss of calcium-sensitivity has occurred, this might still persist after resolution of renal failure such as following renal transplantation (13).

#### 3.1.2 Familial hypocalciuric hypercalcemia (FHH)

Another rare condition of elevated serum calcium levels is FHH, which is inherited in an autosomal dominant pattern and has an estimated prevalence in the range of 1:10,000–1:100,000 (39). The mutations in the calcium-sensing receptor (CaSR) gene result in a reduced sensitivity of parathyroid and renal cells to calcium levels. A higher threshold for calcium to suppress PTH and to reduce tubular calcium reabsorption is therefore required. Laboratory findings typically reveal mild hypercalcemia, inadequately normal PTH levels, and significant hypocalciuria with a calcium-creatinine clearance ratio (CCCR) of less than 0.01 (39). Usually, FHH is asymptomatic and remains uneventful during pregnancy. Again, newborns are at high risk of severe hypocalcemia and seizures during the first days as maternal hypercalcemia inhibits fetal PTH secretion (40). Neonatal hypoparathyroidism can even persist for several weeks (41). Pregnant women with FHH should be identified, which is challenging. Increased intestinal calcium absorption in pregnancy causes absorptive hypercalciuria while hypocalciuria is expected in FHH (9). Furthermore, hypercalcemia exceeding 3 mmol/L was observed during gestation in a patient with FHH (42), as PTH is not suppressed in both PHPT and FHH. As laboratory studies may not be helpful in differentiating these two conditions in pregnancy, imaging procedures and possibly genetic testing may be offered (10, 39), especially to avoid unnecessary parathyroidectomy (42). The preselection of patients at risk is a challenge.

### 3.2 PTH-independent causes of gestational hypercalcemia

PTH-independent disorders include sarcoidosis and other granulomatous diseases, drug- and nutritive-induced hypercalcemia including calcium alkali syndrome (CAS), CYP24A1 mutations, and PTHrP- and malignancy-related hypercalcemia.

#### 3.2.1 Sarcoidosis and other granulomatous diseases

Sarcoidosis is a granulomatous multisystemic disease, which typically occurs in childbearing age. Its manifestations range from slight abnormalities on chest radiology to progressive multiorgan failure (43). Pulmonary sarcoidosis is the most common presentation (affecting more than 90% of cases), but other organ involvements may also occur, including cardiac, renal, hepatic, neurological, ocular, cutaneous, and musculoskeletal systems. Prevalence of gestational sarcoidosis was found to be 0.01% (9.6/100,000) in a retrospective study with more than 7 million deliveries (44). Based on older figures, an incidence of 0.02–0.06% was reported in another publication (45). There are conflicting data on the course of sarcoidosis in pregnancy and on their interaction with gestation (45, 46). Usually, stable disease or even remission is observed (47). Clinical, laboratory, and radiological improvement of sarcoidosis was reported in several pregnant women who confirmed a regression of their usual complaints. It was proposed that the shift from a Th^1^- to a Th^2^-weighted immune response occurring in pregnancy may lead to an increase in anti-inflammatory cytokines and improvement of disorders characterized by a Th^1^-response such as sarcoidosis (45, 47). Additionally, elevated serum cortisol level due to enhanced endogenous production may ameliorate the sarcoidosis in pregnancy (46, 47). However, sarcoidosis significantly increases the risk of venous thromboembolism, hypertensive disorders of pregnancy, FGR, prematurity, and postpartum hemorrhage (44, 47, 48). Notably, the prevalence of preexisting diabetes and hypertension, which is higher in gestational sarcoidosis, may impact these outcomes as well (48). Cardiac involvement, which occurs in 25–50% of patients, is associated with the worst prognosis (43, 47). Life-threatening presentations of cardiac sarcoidosis include recurrent syncope due to ventricular tachyarrhythmia or high-grade atrioventricular block, and heart failure from cardiomyopathy (43, 49). Maternal sudden death may rarely occur and is usually due to cardiac or neurosarcoidosis (47). Not surprisingly, the diagnosis of sarcoidosis may be found after atypical presentations such as granulomatous mastitis or miliary sarcoidosis (50, 51).

Hypercalcemia and hypercalciuria are common laboratory findings in sarcoidosis, while PTH is usually suppressed. An increase in serum calcium levels may even be the first sign of sarcoidosis (52). In pregnant patients with sarcoidosis, severe hypercalciuria may occur, which is mainly due to elevated calcitriol levels based on both extrarenal 1α-hydroxylase activity and a naturally occurring twofold enhanced renal 1,25(OH)_2_D production in pregnant women (9). Granuloma-forming macrophages produce 1α-hydroxylase in sarcoidosis, leading to this extrarenal synthesis of calcitriol. As a result of rising 1,25(OH)_2_D levels, hyperabsorptive hypercalciuria with a consecutively increased renal calcium excretion occurs. Furthermore, calcitriol can suppress PTH and may enhance hypercalciuria by reducing tubular calcium reabsorption (53).

We have to acknowledge that gestational sarcoidosis is a diagnosis of exclusion (47). In pregnancy, invasive diagnostic options are limited due to fetal risks. Noninvasive diagnostic markers, such as serum angiotensin-converting enzyme (ACE) levels, may be altered during gestation and are therefore unreliable for monitoring sarcoidosis (47). The large differential diagnosis of granulomatous diseases includes infections such as tuberculosis, leprosy, histoplasmosis, coccidioidomycosis, and cat-scratch disease (13). Among the non-infectious conditions are polyangiitis granulomatosis, Crohn’s disease, and silicon- or paraffin-induced granulomatosis.

#### 3.2.2 Drug- and nutritive-induced hypercalcemia

In the general population, excessive oral or parenteral vitamin D intake represents the third cause of hypercalcemia (54). Typical manifestations of vitamin D toxicity include hypercalcemia and hypercalciuria, which may be complicated by nephrolithiasis (12). Vitamin D intoxication in pregnancy is rare but mild. Hypercalcemia, hypervitaminosis D and nephrocalcinosis may occur already after moderate vitamin D application in pregnancy (55, 56). Neonatal morbidities resulting from this condition, such as hypercalcemia with bradycardia, have been reported (55). Since CYP24A1 activity inactivates vitamin D, in certain cases the diagnosis of loss-of-function mutations of CYP24A1 was made (57). Nevertheless, in general administration of vitamin D doses up to 5,000 UI daily did not lead to adverse maternal or infant outcomes, nor did it cause alterations in serum levels of calcium, PTH, or calcitriol (9). Doses as high as 200,000 UI per day, most often started in the second trimester of pregnancy, are usually not associated with adverse fetal effects (12).

Next, to vitamin D, drug-induced gestational hypercalcemia (and acute interstitial nephritis) were reported after omeprazole intake (58). Furthermore, vitamin A excess induces osteoclast activity resulting in increased bone resorption and hypercalcemia (59). Other medications known to potentially cause hypercalcemia include lithium, thiazide diuretics, tamoxifen, aromatase inhibitors, theophylline, foscarnet, TNFα-antagonist and PTH analogues. Lithium typically causes parathyroid hyperplasia but is rarely used during pregnancy due to its well-known teratogenicity (13).

Excessive calcium intake during pregnancy has comparable effects to those observed in PHPT, including increased calcium absorption with maternal hypercalcemia and elevated calcium transfer to the fetus with suppression of the fetal parathyroid glands (9). The rare cases of elevated calcium ingestion as the source of pregnancy-associated hypercalcemia are mostly due to CAS (60), former known as milk-alkali syndrome (61). CAS is characterized by the triad of elevated calcium levels, metabolic alkalosis, and acute kidney injury. CAS results from overconsumption of calcium containing products (usually more than 2.5–3 g per day) and intake of alkaline substances such as certain antacids which may be used in self-medication of the frequent gastroesophageal reflux disease in pregnancy (60). Multiple renal factors contribute to maintain CAS (62), which may lead to life-threatening hypercalcemia together with the physiologically increased calcium absorption in pregnancy. Reported complications of CAS include nephrolithiasis, pancreatitis, seizures, and hypertensive disorders of pregnancy (61, 63). Finally, neonatal hypoparathyroidism with hypocalcemic seizures were reported in women with a calcium intake of 3–6 g daily (64).

#### 3.2.3 Hypercalcemia due to loss-of-function mutations in CYP24A1

Inactivating mutations in CYP24A1, which encodes the 24-hydroxylase responsible for the inactivation of vitamin D metabolites, may result in decreased levels of 24,25-dihydroxyvitamin D (24,25(OH)_2_D), accumulation of 25(OH)D and calcitriol, and hypercalcemia. It has been suggested that these mutations occur in approximately 1:32,000 births (65). Diagnosis is based on an increased 25(OH)D to 24,25(OH)_2_D ratio and can be confirmed by genetic testing. To date, more than 20 pathogenic variants have been described. Most patients have homozygote or compound heterozygote mutations. Though it is a hereditary disease, vertical genetic transmission to the child with consecutive neonatal hypercalcemia is conceivable. While most of the patients are asymptomatic, pregnancy may trigger the development of hypercalcemia due to physiological upregulation of calcitriol and the widespread vitamin D and calcium supplementation during gestation (66). Maternal and obstetric complications occur frequently, involving pancreatitis, nephrolithiasis, altered mental status, impaired kidney function, hypertensive disorders of pregnancy, and fetal loss (65, 67). Neonatal complications are rare (65) but may include hypoglycemia due to hypercalcemia-induced hyperinsulinemia (68).

#### 3.2.4 Parathyroid hormone related protein (PTHrP) and malignancy-related hypercalcemia

PTHrP-induced hypercalcemia, the so-called humoral hypercalcemia, may occur during pregnancy, which is normally due to an excessive physiologic release of PTHrP from the mammary or placental tissue (9). PTHrP exhibits a high degree of homology to the parathyroid hormone and has a similar affinity for the PTH receptors. Therefore, PTHrP-mediated hypercalcemia is also referred to as pseudohyperparathyroidism. PTHrP has a half-life of minutes in the circulation. In consequence, an abrupt drop in serum calcium is expected after delivery in cases where the placenta is the site of PTHrP overproduction. This was observed in a patient who presented with a hypercalcemic crisis (5,24 mmol/L), markedly increased PTHrP and suppressed PTH in the third trimester. In these cases within a few hours following delivery, serum calcium is normalized and PTHrP is undetectable (69, 70). Another rare source of elevated PTHrP in pregnant women may be breast tissue resulting in hypercalcemia and only resolving after a bilateral mastectomy during pregnancy (71, 72), termination of pregnancy (73), weaning (74) or successful treatment with a dopamine agonist (75). Notably, nephrocalcinosis, acute pancreatitis, and osteoporotic fractures are detected in PTHrP-related hypercalcemia during pregnancy as well (9, 75, 76).

While malignancy is the second most common etiology of hypercalcemia in the general population (54), it is rarely diagnosed in pregnant women. The underlying cause is primarily a pathological PTHrP-release, leading to increased bone resorption and renal calcium reabsorption (9). Other etiologies of malignancy-associated hypercalcemia include osteolytic bone metastases (skeletal lysis by tumor activity-induced secretion of cytokines such as interleukin (IL)-1, IL-6, and tumor necrosis factor), excessive extrarenal calcitriol formation (activity of 25(OH)D 1α-hydroxylase expressed in tumor cells), and rarely ectopic formation of PTH by tumoral cells (13). Reported cases of malignancy-related hypercalcemia in pregnancy include multiple myeloma (77), cholangiocarcinoma (78), neuroendocrine pancreatic tumor (79, 80), renal (81) and ovarian carcinomas (82, 83), choriocarcinoma (84), and metastatic breast carcinoma (85). The resulting chronic hypercalcemia in the mother usually leads to fetal hypoparathyroidism with a high risk of severe neonatal hypocalcemia. Benign tumors contributing to critical gestational hypercalcemia are rare (14, 86, 87).

#### 3.2.5 Other causes of hypercalcemia

Additional uncommon causes of hypercalcemia in pregnancy or the early postpartum period have been observed in several cases, mainly endocrinological diseases. Lymphocytic hypophysitis with secondary adrenal insufficiency and hyperthyroidism due to thyroiditis was reported (88). It was supposed that increased thyroid activity may lead to increased osteoclastic activity complicated by hypercalcemia (13). In adrenal insufficiency, increased bone resorption, likely attributed to the action of thyroid-stimulating hormone (TSH) resulting from the loss of inhibition by glucocorticoids (GC), and decreased renal calcium excretion may lead to elevated serum calcium levels (13). Furthermore, pheochromocytoma was linked to hypercalcemia in a pregnant woman (89). Finally, immobilization due to reduced physical activity and bedrest during gestation may contribute to bone resorption and therefore hypercalcemia (9).

## 4 Clinical presentation and complications

The clinical manifestations of gestational hypercalcemia is often nonspecific and can be easily mistaken for common pregnancy-related complaints. These typically include gastrointestinal symptoms such as nausea, vomiting, and constipation, but also asthenia, muscle weakness, difficulty concentrating, and weight loss (9, 14). Hypercalcemia can even mimic hyperemesis gravidarum (90). Other nonspecific symptoms include skeletal pain, particularly arthralgias, or unusual headaches (13, 14). Symptoms like anxiety and depression may be exacerbated by pregnancy-related stress (6) and in rare cases seizures were reported (91).

**Figure 2:**
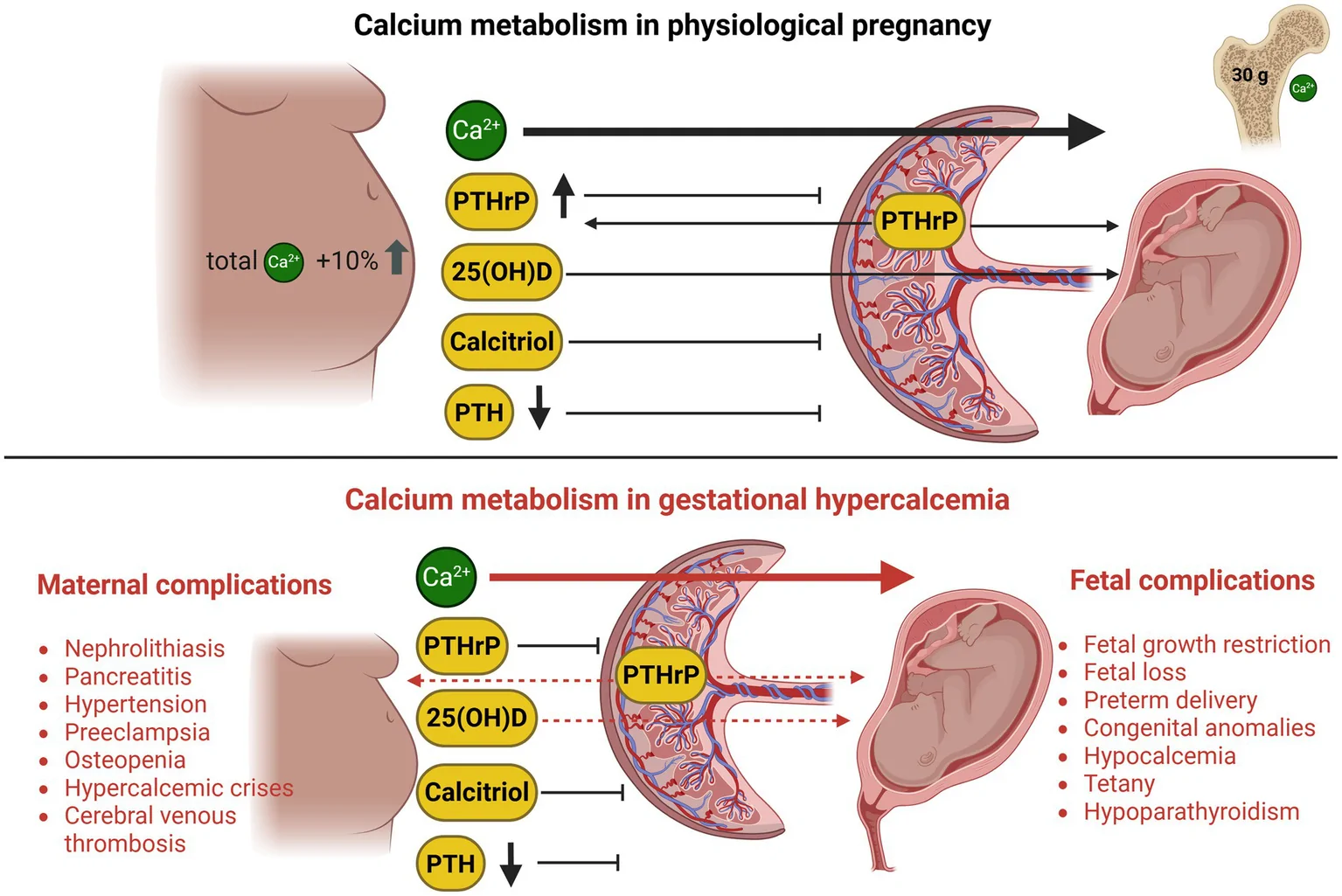
Impaired calcium metabolism in gestational hypercalcemia. In physiological pregnancies maternal serum calcium (Ca^2+^) passes the placenta to the fetus who lacks an independent calcium source. PTHrP is produced by several tissues, including the placenta. Placental PTHrP can reach the fetus. PTHrP partially suppresses maternal PTH during pregnancy and PTH does not cross the placenta. Maternal 25(OH)D is transmitted to the fetus, playing a crucial role in fetal vitamin D status, as the active form, calcitriol, does not cross the placenta and the fetal kidneys only start to produce calcitriol in the third trimester. In gestational hypercalcemia maternal serum calcium levels are elevated, resulting in pathologies affecting both mother and fetus. Elevated calcium concentration lead to partial suppression of PTHrP and 25(OH)D, the latter crossing the placental barrier, causing a vitamin D shortage for the fetus. Maternal complications of hypercalcemia include nephrolithiasis, pancreatitis, hypertension, preeclampsia, osteopenia, hypercalcemic crises, and cerebral venous thrombosis. Fetal complications of gestational hypercalcemia include fetal growth restriction, fetal loss, preterm delivery, congenital anomalies, hypocalcemia, tetany and hypoparathyroidism. PTHrP, parathyroid hormone related protein; PTH, parathyroid hormone.

Hypercalcemia usually results in hypercalciuria, which activates renal CaSR (62). This induces renal resistance to vasopressin leading to polyuria and hypovolemia. Therefore, hypercalcemic patients typically present with dehydration (14), which should result in hypotension. However hypercalcemia additionally affects vascular tone as well and therefore the blood pressure does not reflect a true volume status (13). Besides volume depletion in the context of hypercalciuria-related polyuria, other mechanisms such as reduced renal blood flow due to vasoconstriction of the afferent arteriole and tubular obstruction by calcium precipitates contribute to the risk of acute renal failure (62). Additional renal complications include nephrocalcinosis and -lithiasis (14). Finally, longstanding maternal hypercalcemia may result in arterial hypertension and pancreatitis (33, 65, 90) contributing to obstetrical complications such as fetal loss, FGR, PE, and preterm delivery (31, 92) as shown in Figure 2.

Clinical relevance and symptoms of gestational hypercalcemia may vary. Mild gestational hypercalcemia usually remains asymptomatic and the diagnosis is not present or made incidentally (8, 11, 24, 25). In particular, serum calcium levels below 2.8 mmol/L may go undetected (13). However, a corrected serum calcium level of 3.5 mmol/L constitutes an acute hypercalcemic crisis and is associated with multiorgan dysfunction (13). Besides already above mentioned acute renal injury (60), neurologic manifestations such as confusion and stupor (72, 76) and acute respiratory distress syndrome were reported (93). Furthermore, severe cardiac manifestations, such as high-grade atrioventricular block, malignant arrhythmias, and cardiac arrest, may occur (13).

Gestational hypercalcemia contributes to adverse neonatal outcomes by directly affecting the fetus (Figure 2). For example, gestational hypercalcemia may lead to fetal polyuria by elevating fetal calcium levels (8, 94). A further consequence of chronic maternal hypercalcemia with excessive transplacental calcium transfer is the suppression of the fetal parathyroid glands, namely resulting in fetal hypoparathyroidism. Consequently, after delivery, when the maternal calcium supply is abruptly terminated, the newborn is at a high risk of developing severe neonatal hypocalcemia which may be complicated by tetany and even death (14, 95). In line with this notion, hypercalcemia in the newborn has been reported (65, 79) and older studies report high fetal mortality rates associated with moderate to severe maternal hypercalcemia (13).

## 5 Diagnostic options

**Figure 3:**
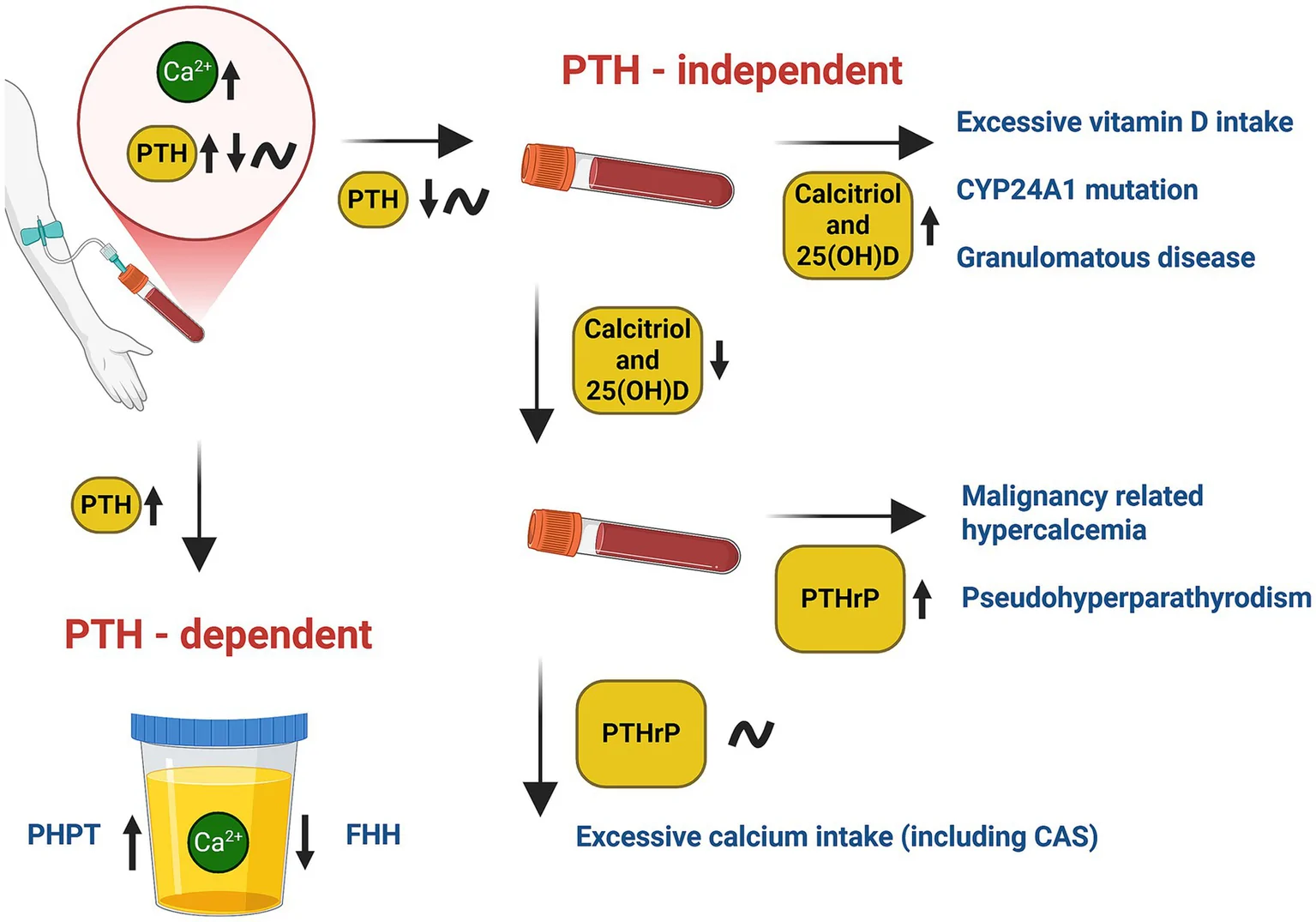
Etiology and diagnostic steps of gestational hypercalcemia. Hypercalcemia is diagnosed by high maternal serum calcium (Ca^2+^) levels and PTH—dependent or—independent causes may be considered. Further diagnostic steps involve may involve measuring maternal urinary calcium (Ca^2+^) and further serum testing, including calcitriol and 25(OH)D. The diagnostic flowchart points towards the most likely diagnosis. PTHrP, parathyroid hormone related protein; PTH, parathyroid hormone.

The most common laboratory findings of hypercalcemia in pregnancy are summarized in Figure 3. The diagnosis is based on an increase in ionized (and for screening purposes only) serum calcium levels. Secondly, the parallel measurement of PTH and calcium levels is essential to distinguish PTH-dependent from PTH-independent disorders. Routine testing involves urinary calcium excretion, which is usually assessed by using the CCCR. As renal impairment is a common finding in the presence of hypercalcemia and -calciuria, serum levels of creatinine, urea, and electrolytes should be included in the laboratory workup (21). Moreover, it should be completed by the remaining parameters of calcium metabolism, including phosphate and liver enzymes. If hereditary causes of gestational hypercalcemia are suspected, genetic testing might be indicated (20, 22).

### 5.1 PTH-dependent diagnostic options

The detailed diagnosis of gestational PHPT includes imaging cervical sonography with operator-dependent sensitivity and specificity of 69–87% and 94–96%, respectively (8, 13). Additionally, ultrasound may also be employed to exclude nephrocalcinosis and kidney stones as frequent complications of hypercalcemia. If surgical removal of abnormal parathyroid glands is considered, localization should be confirmed by a second imaging method. Ionizing radiation can be avoided by cervical magnetic resonance imaging (MRI) (1, 96), which also has a sensitivity and specificity >97% (13). Formally contraindicated during pregnancy to fetal radioactive exposure, ^99m^Tc-MIBI scan and ^18^F-fluorocholine positron emission tomography/computer tomography (PET/CT) may still be used safely in selected women (1). Fetal exposure of less than 5 mGy did not appear to be associated with an increased risk of pregnancy complications or birth defects (8, 97). Also, a case of a pregnant patient with symptomatic hypercalcemia and suspected recurrence of parathyroid carcinoma was reported, in whom sonography and MRI failed to localize the culprit lesion. PET/CT was therefore successfully performed during the third trimester, allowing surgical excision of the malignant tumor (98). However, given the lack of long-term fetal data, any kind of radiological examination during pregnancy should be avoided if possible.

Fine needle aspiration with cytologic analysis may be helpful in differentiating a parathyroid mass from other pathological lesions in the neck such as thyroid nodules or lymph nodes. Depending on the woman’s symptoms and suspected complications of hypercalcemia, further organ specific paraclinical examinations are indicated, such as bone densitometry, echocardiography, and a MRI scan of the chest and abdomen (70).

### 5.2 PTH-independent diagnostic options

If PTH is suppressed or normal, a wide range of possible causes have to be considered. 1,25(OH)_2_D and 25(OH)D serum levels are measured if vitamin D-mediated etiologies of hypercalcemia such as granulomatous disease or vitamin D intoxication are suspected. It is essential to note that a calcitriol excess may be overlooked if only a low 25(OH)_2_D is measured, since an elevated 1 *α* -hydroxylase is shifting 25(OH)D to 1,25(OH)_2_D. Furthermore, PTHrP may be useful to confirm or rule out pseudohyperparathyroidism. Timely analysis might yet be a challenge as only highly specialized laboratories are offering this test. Depending on the suspected diagnosis, specific laboratory tests are indicated, such as TSH, 25(OH)D to 24,25(OH)_2_D ratio, *γ*-interferon, or vitamin A.

## 6 Therapeutic options

### 6.1 General measures

Managing hypercalcemia in pregnant women is challenging as no randomized controlled trials exist. All recommendations base on observational studies and expert opinions (1). Ideally, a causal therapy of hypercalcemia should be preferred if the origin is known and treatable but fetal drug exposure has to be considered. The primary goal is to reduce serum calcium levels and several options exist. Intravenous volume replacement to correct hypovolemia present in patients with hypercalciuria-induced polyuria and to enhance renal calcium excretion by increasing diuresis is particularly important (61). If only mild hypercalcemia occurs and the women can be managed in an outpatient setting, oral rehydration may be sufficient (11). Dietary calcium intake should be limited, and any calcium-containing supplements stopped. Hydration and low-calcium diet may be sufficient to decrease serum calcium levels and relieve symptoms in pregnant women with PHPT (35). We summarized the most common therapeutics according to the diagnosis in Table 1.

**Table 1:** Most common diagnosis and therapeutic options diagnosis.

Therapeutic options	Primary hyperparathyroidism	Granulomatous disease (including sarcoidosis) and vitamin D excess	High calcium intake or supplementation	Familial hypocalciuric hypercalcemia	PTHrp−/malignancy-related hypercalcemia
Oral rehydration/i.v. volume replacement	✓	✓	✓	✓	✓
Calcium intake restriction/stop of calcium-containing supplement	✓	✓	✓	✓	✓
Stop of vitamin D-containing supplement	*	✓	✓	*	✓
Loop diuretics	✓	✓	✓	✓	✓
Calcitonin	✓	✓	✓	✓	✓
Bisphosphonates	(✓)	(✓)	(✓)		(✓)
Denosumab	CI	CI	CI		CI
Cinacalcet	✓			✓	
Glucocorticoid		✓			
Hydroxychloroquin/azathioprine/biologics		✓ (in sarcoidosis)			
Surgical or interventional therapy (parathyroidectomy)	✓				

✓, considered safe in pregnancy; (✓), safety concerns for use in pregnancy; CI, contraindicated in pregnancy; *, depending on maternal vitamin D status.

#### 6.1.1 Calcium-lowering therapy

Multiple therapeutic options exist and choice of a drug depends on the origin of hypercalcemia. Loop diuretics may be administered to enhance diuresis and calciuria by inhibiting the natrium-kalium-cotransporter 2 (NKCC2) channel in the Henle loop, which finally leads to a decrease in tubular calcium reabsorption. In contrast, thiazide diuretics should be avoided, as they enhance proximal renal calcium reabsorption and thus increase serum calcium levels. Other drugs that may contribute to hypercalcemia such as lithium or vitamin D should be reevaluated carefully. For the latter, cautiously ensuring maternal 25(OH)D levels in the normal range should be advised as this may decrease PTH level in PHPT and provide a sufficient vitamin D status in the newborn, which in turn may limit the risk of postnatal hypocalcemia (11).

#### 6.1.2 Inhibition of osteoclastic activity

Calcitonin mainly reduces serum calcium levels by inhibiting osteoclastic activity and enhancing renal calcium excretion. It does not cross the placental barrier and has been used safely in several cases of hypercalcemia in pregnant patients (1, 14, 99). However, calcitonin has a time-limited efficacy given the rapid tachyphylaxis with downregulation of its receptors on osteoclasts occurring after a few days (65, 100). Therefore, the maximally observed decrease in serum calcium was 0.3–0.5 mmol/L only.

Bisphosphonates (BP) have a stronger calcium-lowering effect than calcitonin. BP inhibit osteoclast activity and decrease calcium release from the bones due to their very long skeletal half-life. They cross the placental barrier and therefore recommendations argue against the use of BP in pregnant women (1). These recommendations are mainly due to teratogenic effects such as a decrease in fetal bone length (observed in animals) or association with congenital malformations (in humans up to 20% of cases in the past) (101). However, a recent systematic review including 108 pregnancies exposed to BP did not confirm the significantly increased risk of either obstetrical complications, such as miscarriage, low birth weight, and preterm birth, or neonatal hypocalcemia, while the rate of congenital malformations was only slightly increased (102). Several cases, where hypercalcemia was successfully treated with intravenous BP in pregnant women, have been reported. For example, pamidronate was used during the last trimester in two patients with metastatic breast cancer (85, 103). There were no observed adverse effects in the infant. A pregnant woman with cholangiocarcinoma-induced hypercalcemia was also treated with pamidronate, which normalized maternal calcium levels (78). The newborn was delivered prematurely by caesarean section due to worsening of the fetal condition, but no adverse outcome associated with BP therapy was reported. A case of suspected excessive placental PTHrP with hypercalcemia, which did not respond to conservative treatment with isotonic saline, loop diuretics and calcitonin, was treated with pamidronate in the third trimester (70). The neonate suffered from hypocalcemia. In another woman with CAS, etidronate was administrated in the second trimester (104).

Finally, denosumab also inhibits the osseous calcium release, but crosses the placenta and is teratogenic. It should therefore be avoided during pregnancy (8).

#### 6.1.3 Modulation of parathyroid sensitivity and calcium metabolism

In hypercalcemic PHPT during pregnancy, calcium-reducing therapy with cinacalcet may be considered, although it does cross the placenta (1). Cinacalcet is a calcimimetic agent that increases the sensitivity of parathyroid CaSR to extracellular calcium and thus reduces PTH levels. CaSR is also expressed in other tissues including the placenta, and cinacalcet could thus theoretically influence the placental calcium transfer (97), but to date, no teratogenicity was described. However, cinacalcet use in pregnancy has only been reported in a few cases including PHPT due to parathyroid adenoma (14, 98, 105), parathyromatosis (106), and parathyroid carcinoma (107). A recent publication found a least 17 cases of women with gestational PHPT due to benign etiology who were treated with cinacalcet (108). Safety evidences for the use in pregnant patients are thus still insufficient for official approval (1). A common adverse effect is nausea and vomiting (14, 97, 108) and neonatal hypocalcemia occurred in some cases (8, 107). Again, whether it is related to the use of cinacalcet in pregnancy or to maternal hypercalcemia itself is uncertain (8). Successful use of cinacalcet has also been reported in FHH-induced symptomatic gestational hypercalcemia (39).

Corticosteroids are mainly used, if hypercalcemia is caused by vitamin D intoxication or granulomatous diseases such as sarcoidosis with increased formation of 1,25(OH)_2_D. GC inhibit 1α-hydroxylase activity, resulting in a reduced calcitriol production and therefore a decrease in intestinal calcium absorption. Passage of GC from the maternal to the fetal circulation is regulated by placental 11β-hydroxysteroid dehydrogenase type 2, which is responsible for the conversion of GC into a biologically inactive form. In pregnancy, non-fluorinated GC such as prednisone and hydrocortisone are preferred due to their limited transplacental passage and the use at the lowest effective dose is recommended (109). Hydrocortisone is the least potent GC and mostly metabolized in the placenta (110). There are conflicting data regarding the association between the use of GC during pregnancy and the risk of premature birth, low birth weight, PE, and gestational diabetes (111). The use of corticosteroids is widely accepted, especially if dosing does not exceed 100–150 mg hydrocortisone equivalent.

#### 6.1.4 Macronutrients and others

Different macronutrients, such as intravenous magnesium has been used previously (112, 113). It is supposed to lower serum calcium by activating the CaSR, which also detects non-calcium divalent cations. Oral phosphate-containing drugs, which may decrease intestinal calcium absorption, were employed in the treatment of gestational hypercalcemia as well (112). Given the increased risk of intra- and extravascular calcium-phosphate deposition, resulting in soft tissue calcification and organ failure or and diarrhea (112, 114), they are now rarely used (65, 99). Furthermore, a case report was published that described the use of oral potassium supplementation (potassium dihydrogen phosphate) due to its potential to reduce intestinal calcium absorption (65). Given the potential of other calcium lowering agents (see above), macronutrients use is rather limited.

Azole agents (ketoconazole and fluconazole), inhibiting CYP27B1 and thus 1α-hydroxylase responsible for the conversion from 25(OH)D to 1,25(OH)_2_D, have been documented as efficacious treatments for hypercalcemia due to CYP24A1 mutations in nonpregnant patients (67). Another drug, which has been reported to lower calcium successfully in this condition, is rifampicin. It degrades vitamin D metabolites by inducing CYP3A4 (67). As fluconazole and rifampicin have been employed safely in treating infection during pregnancy, they may therefore be considered for the treatment of gestational hypercalcemia (65). If hypercalcemia is markedly elevated and unresponsive to medical treatment, or presents with severe symptoms, extracorporeal calcium elimination by hemodialysis may be indicated (23, 60, 93). However, renal replacement may cause hypotension leading to fetal distress, and reduce serum levels of gestational hormones such as progesterone potentially resulting in uterine contractions (13).

#### 6.1.5 Surgical and interventional therapy

The only causal treatment approach for PHPT is surgical removal. Parathyroidectomy in pregnancy was associated with a significantly lower fetal complication rate compared to conservative therapy (9.1% versus 38.9%) in a systematic review including 382 women with gestational PHPT, which also applied to asymptomatic cases (115). Similar results were found in a more recent review regarding adverse neonatal outcomes in surgical versus non-surgical treatment of gestational PHPT (10% vs. 49.6%) (116). Adverse effects were less likely to occur following surgery during the second trimester of pregnancy than in the third one (4.5% vs. 21.1%) (115). An older case series reported no maternal or neonatal surgical and delivery complications among women who underwent parathyroidectomy during the second trimester (31). Furthermore, it is likely that the risk of anesthesia and surgery in pregnant women is the lowest in the second trimester with regard to general complications, premature birth, and neonatal mortality (9). According to this, the European expert consensus on parathyroid disorders recommends to perform parathyroidectomy in the second trimester of pregnancy in women with PHPT and albumin-corrected serum calcium level greater than 2.85 mmol/L (and/or above 0.25 mmol/L of upper limit of normal (ULN) and/or ionized calcium greater than 1.45 mmol/L) (1). Notably, hypercalcemia greater than 2.85 mmol/L is associated with an increased risk of miscarriage (31). The Canadian and international consensus on PHPT also favor parathyroidectomy during the second trimester if ionized calcium is above 0.12 mmol/L of upper limit of normal (2). However, surgical removal in the last trimester is also feasible and should be considered if necessary (31). In line, successful parathyroidectomy during the first trimester in women with persistent symptomatic hypercalcemia despite conservative treatment were reported (100, 117).

The preferred surgical approach in pregnant patients is minimally invasive parathyroidectomy (MIP) with intraoperative PTH monitoring (1), but surgical neck exploration may be needed as several imaging methods are contraindicated in pregnancy (11). Cervical plexus block is a feasible alternative to general anesthesia during pregnancy (35). If surgery is postponed, it is advisable to plan parathyroidectomy after delivery, at the latest before a subsequent pregnancy (118). FHH should be ruled out before parathyroidectomy as surgical treatment is not indicated in this case (42). Recently, thermal ablation technics of parathyroid adenoma have been used safely in several pregnant women. Effective microwave ablation in two cases of symptomatic gestational PHPT has been performed (29, 119). Also, a few patients have been treated with radiofrequency ablation in the second trimester (13). These ultrasound-guided percutaneous thermal ablation strategies seem to be an interesting alternative curative treatment option of PHPT in pregnancy and may be more explored in the future.

#### 6.1.6 Other etiological approaches

In the context of sarcoidosis, GC represent the primary therapeutic option (47). The majority of the steroid-sparing immunosuppressive agents including methotrexate, leflunomide and mycophenolate are teratogenic and therefore, their use in pregnancy is limited (109). Among second-line therapies for sarcoidosis, hydroxychloroquine is safe and additionally some evidence for azathioprine exist (45, 109). The use of biologics could be a safe third-line alternative in the management of sarcoidosis in pregnancy (47). Given the increased risk of PE and FGR, prophylactic aspirin application may be considered (47). Notably, calcium and vitamin D supplementation should be avoided given the elevated risk of hypercalcemia in sarcoidosis (47).

## 7 Discussion

Gestational hypercalcemia is rare but of relevance and commonly underdiagnosed. The most common cause of gestational hypercalcemia is PHPT (over 90% of cases), which in turn is due to single parathyroid adenoma in more than 80%. In general, causes of gestational hypercalcemia can be divided into PTH-dependent etiologies, with increased or inappropriately high PTH levels, mainly PHPT and FHH, versus PTH-independent disorders (see Figure 2). Extended laboratory work-up is the most useful paraclinical examination in gestational hypercalcemia. This is of importance as hypercalcemia and elevated PTH values in pregnant women have both been related to hypertensive disorders (10, 32, 65). In retrospective studies, PE was diagnosed in up to 15–30% of patients with gestational PHPT (17, 114), compared with around 5% in the general population. Furthermore, parathyroid adenoma was found to be significantly associated with subsequent PE (odds ratio 6) (32). Interestingly, the increased risk for PE persisted even for up to 5 years following successful treatment of the adenomas, which makes the postpartum evaluation of patients relevant. Additionally, the association between severity of PHPT-induced increase in maternal calcium levels and risk of PE was suggested (114) and gestational hypercalcemia resulting from causes other than PHPT have been related to maternal hypertension and PE (63, 65, 79, 87). Together, these findings support the state of hypercalcemia as a possible independent risk factor for hypertensive disorders in pregnant women (120).

The sympathetic nervous system and the renin-aldosterone axis may additionally have a role in the pathogenesis (10). In nonpregnant individuals, heightened peripheral vascular resistance was observed in acute hypercalcemic hypertension, accompanied by increased serum epinephrine levels. The latter was proposed to play a contributory role (121). Furthermore, elevated serum levels of PTH and calcium were supposed to increase endothelial damage and cellular calcium influx, insulin resistance, and cardiovascular disease, and therefore the risk of PE (19, 32). Also, worsening of renal function secondary to hypercalcemia and hypercalciuria may be implicated in the development of hypertensive disorders.

Delays in the diagnosis of gestational hypercalcemia are frequently observed due to an asymptomatic course or an overlap with usual gestation-related clinical manifestations (17, 32). Even in women with documented hypercalcemia prior to conception, nearly 50% of PHPT were diagnosed in the postpartum (5). In instances where symptoms likely attributable to pregnancy, including nausea, emesis, constipation, and fatigue, persist, or where complications, such as hypertension, pancreatitis, and nephrolithiasis, occur, measurement of albumin-corrected or ionized calcium is indicated to rule out hypercalcemia. Finally, unexplained arterial hypertension during the early gestation period should be investigated for secondary causes, including hypercalcemia and PHPT (10). This is of importance as early treatment reduces the risk of adverse maternal and infant outcomes.

Current research does not support the widespread theory that low protein intake is associated with an increased risk of PE, and studies did not show any decrease in the incidence of PE in women with protein supplementation during pregnancy (122). Therefore, high protein supplementation is not recommended in pregnant individuals to improve maternal and perinatal outcomes (123). In pregnant women with low dietary calcium intake, calcium supplementation is effective in preventing PE as a 64% risk reduction has been reported in certain environmental conditions (123). Therefore, a daily calcium supplementation of 1.5–2 g during pregnancy is advised in populations with a low calcium diet (124). In contrast, no beneficial effect of calcium supplementation was observed in pregnant patients with an adequate dietary calcium intake (9). In regard to supplementation of vitamin D in pregnancy, a recent Cochrane systematic review showed no significant effect on the incidence of PE and the majority of other maternal and infant complications (12). Currently, the world health organization (WHO) argues against routine vitamin D supplementation during gestation (125).

Therapeutic options of gestational hypercalcemia consist of observational, medical or surgical treatment (see Table 1). Multidisciplinary approach is always necessary. Mild oligosymptomatic hypercalcemia may be managed by watchful waiting or conservative therapy. Laboratory monitoring of the serum calcium and renal function at least every four weeks is recommended as in our presented cases (1, 21). There are no uniform guidelines for the management of gestational hypercalcemia, but it primarily involves intravenous or oral rehydration. In several cases, it may be combined with loop diuretics. There is no official approval for calcium-lowering treatments, but calcitonin and cinacalcet have been used safely during gestation. A combination of calcitonin and cinacalcet was found to be effective in some patients but was usually associated with significant nausea (14, 105). GC such as hydrocortisone or prednisone are usually safe in gestation and may be used successfully in cases of hypercalcemia associated with granulomatous disease with increased 1,25(OH)_2_D levels (109, 110).

The European expert consensus on parathyroid disease recommends to perform parathyroidectomy in cases of PHPT with albumin-adjusted hypercalcemia greater than 2.85 mmol/L in gestation, as this level is related to an increased complication rate (1). In some conditions such as acute pancreatitis or prior unexplained pregnancy loss, surgical treatment may yet be indicated at lower calcium levels (greater than 2.7 or 2.75 mmol/L respectively) (126, 127). The available data even suggest that parathyroidectomy should be considered in all pregnant women with PHPT regardless of symptomatology, as surgical therapy is associated with fewer risks and better infant outcomes than conservative treatment (115). The preferred time of surgery is in the 2nd trimester, as the risk of anesthesia may be higher during the first (incomplete organogenesis) and third (inducing preterm labor) trimester, but it should not be deferred if the risk of hypercalcemia-associated complications is higher than that of parathyroidectomy (11). MIP is the preferred therapeutic option in PHPT in pregnant women due to the advantages of less trauma and shorter operative time with less effect of anesthesia on the fetus (35). In the future, ultrasound-guided percutaneous thermal ablation of parathyroid lesions may become an alternative curative treatment option. Prospective controlled studies to compare therapeutic options of gestational hypercalcemia are ideally needed in the future, but they are difficult to perform. It is a rare condition with different origins and there are several ethical considerations regarding study designs during pregnancy.

According to a European expert consensus, serum calcium concentrations should be measured as part of routine medical screening during preconception and early pregnancy (1), in order to reduce the risk of adverse maternal and fetal outcomes associated with gestational hypercalcemia and its treatment. This suggestion is supported by multiple reviews (3, 13, 65). Despite these recommendations, most of the international guidelines for antenatal care, such as the WHO and the National Institute for Health and Care Excellence (NICE) recommendations including German speaking countries, do not include serum calcium among the screening laboratory tests conducted prior or during gestation (123, 128).

## 8 Conclusion

Hypercalcemia in pregnancy may go undetected or remain uneventful, but can also result in severe maternal, fetal and neonatal morbidity and mortality. Early diagnosis and therapy are needed to avoid complications. Further multidisciplinary investigations are essential as, depending on the etiology, treatment of the underlying cause is possible. If the origin remains unclear, conservative treatment approaches using calcium-lowering medication may be necessary, for which there is still no official approval in pregnancy due to a lack of data. Given the high disease burden of hypercalcemic diseases, screening for hypercalcemia is highly cost effective, if any adverse vital signs such as high blood pressure are observed.
